# Transactions of the American Dental Association

**Published:** 1865

**Authors:** 


### TRANSACTIONS OF THE AMERICAN DENTAL ASSOCIATION.

The transactions of this Association are now published and ready for distribution to the members and others who desire to obtain them. From various causes their issue has been delayed much longer than it should have been.

Tardiness in obtaining the copy was the principle cause, sickness of the secretary was an additional cause.

The papers contained in the transactions are interesting and important, and should be in the hands of every dentist. It is desirable that societies should see to it, that they are properly distributed to those who are not members of the central organization.

